# Current Insights into Weak Seed Dormancy and Pre-Harvest Sprouting in Crop Species

**DOI:** 10.3390/plants13182559

**Published:** 2024-09-12

**Authors:** Angel J. Matilla

**Affiliations:** Departamento de Biología Funcional, Universidad de Santiago de Compostela, 14971 Santiago de Compostela, Spain; angeljesus.matilla@usc.es

**Keywords:** pre-harvest sprouting, seed dormancy, ABA, GAs, α-amylase, domestication, cereals, sprouting resistance

## Abstract

During the domestication of crops, seed dormancy has been reduced or eliminated to encourage faster and more consistent germination. This alteration makes cultivated crops particularly vulnerable to pre-harvest sprouting, which occurs when mature crops are subjected to adverse environmental conditions, such as excessive rainfall or high humidity. Consequently, some seeds may bypass the normal dormancy period and begin to germinate while still attached to the mother plant before harvest. Grains affected by pre-harvest sprouting are characterized by increased levels of α-amylase activity, resulting in poor processing quality and immediate grain downgrading. In the agriculture industry, pre-harvest sprouting causes annual economic losses exceeding USD 1 billion worldwide. This premature germination is influenced by a complex interplay of genetic, biochemical, and molecular factors closely linked to environmental conditions like rainfall. However, the exact mechanism behind this process is still unclear. Unlike pre-harvest sprouting, vivipary refers to the germination process and the activation of α-amylase during the soft dough stage, when the grains are still immature. Mature seeds with reduced levels of ABA or impaired ABA signaling (weak dormancy) are more susceptible to pre-harvest sprouting. While high seed dormancy can enhance resistance to pre-harvest sprouting, it can lead to undesirable outcomes for most crops, such as non-uniform seedling establishment after sowing. Thus, resistance to pre-harvest sprouting is crucial to ensuring productivity and sustainability and is an agronomically important trait affecting yield and grain quality. On the other hand, seed color is linked to sprouting resistance; however, the genetic relationship between both characteristics remains unresolved. The identification of mitogen-activated protein kinase kinase-3 (MKK3) as the gene responsible for pre-harvest sprouting-1 (Phs-1) represents a significant advancement in our understanding of how sprouting in wheat is controlled at the molecular and genetic levels. In seed maturation, Viviparous-1 (Vp-1) plays a crucial role in managing pre-harvest sprouting by regulating seed maturation and inhibiting germination through the suppression of α-amylase and proteases. Vp-1 is a key player in ABA signaling and is essential for the activation of the seed maturation program. Mutants of Vp-1 exhibit an unpigmented aleurone cell layer and exhibit precocious germination due to decreased sensitivity to ABA. Recent research has also revealed that TaSRO-1 interacts with TaVp-1, contributing to the regulation of seed dormancy and resistance to pre-harvest sprouting in wheat. The goal of this review is to emphasize the latest research on pre-harvest sprouting in crops and to suggest possible directions for future studies.

## 1. Introduction

### 1.1. Hormonal Regulation in Embryo-Seed Formation and Seed Dormancy Control

In plants, the zygote undergoes a complex developmental process governed by multiple molecular signals, the mechanism of many of which are largely unknown [1]. The final phase of this zygotic embryogenesis, known as seed maturation, includes the acquisition of desiccation tolerance and primary dormancy, which prevents the seed from germinating, among other singularities [2,3,4]. The intensity of seed dormancy is established during seed maturation, is primarily controlled by ABA/GAs, and dictates the behavior of the seed after shedding or even while still attached to the mother plant [5,6,7,8,9]. Seed dormancy is broken only when the balance shifts from ABA to GAs [10]. Adequate dormancy keeps seeds in a quiescent state, while weak dormancy ensures a higher and more uniform radicle emergence rate after sowing. Several researchers have identified a crucial mechanism that plants use to decide between germination and dormancy, which involves regulating the concentration of ABA. The decision to germinate is a significant gamble for a plant because it determines whether the seed will develop under favorable conditions or risk survival in adverse environments [4,11]. This ABA-regulated pathway is essential for plants to optimize their survival and reproductive success in unpredictable environments.

Dormancy and germination are controlled by numerous genes [12] and quantitative genetic loci (QTLs) (see http://www.gramene.org for the Gramene QTL database) [13,14]. The QTL analysis is indeed a powerful tool in genetic analysis, particularly for studying traits that vary quantitatively and understanding the genetic basis of such traits. The first report of a QTL for cereal grain dormancy was published in the early 1990s [15]. During the second decade of the 21st century, researchers began identifying causal genes (i.e., genes whose variation in sequence leads to changes in the trait of interest) for the major dormancy QTLs of barley and wheat. For example, alanine aminotransferase (*AlaAT*) is the causal gene for the major grain dormancy QTL Qsd1 (SD1) in barley [16], and mitogen-activated protein kinase kinase-3 (*MKK3*) is the causal gene for the major grain dormancy QTL Qsd2-AK (SD2) in barley and PHS1 in wheat [17,18]. Some causal genes underlying significant QTLs have been identified in wheat (*Triticum aestivum*) and their effects on pre-harvest sprouting have been partially characterized, including *TaPHS1* [17,19,20,21], *TaMKK3* [18], *TaVp-1* [22], *TaSdr* [23], and *Tamyb10* [24,25].

Nevertheless, like any other biological process, zygotic embryogenesis can be subject to alterations that deviate from the normal developmental pathway [26]. This is the case with some seeds that fail to enter the normal dormancy period and begin to germinate while still attached to the mother plant, a phenomenon known as pre-harvest sprouting [14]. To prevent high pre-harvest sprouting in crops, researchers have developed a series of molecular alterations. These genetic modifications have been designed to enhance seed dormancy and precisely control the timing of germination, which is essential to avoiding pre-harvest sprouting [12,20,26]. The breakthroughs achieved have been thoroughly discussed and reviewed in the current update, emphasizing the significant progress made in understanding and manipulating the molecular pathways that govern seed dormancy and germination. A good number of alterations in the seed maturation process have been made by humans for their benefit, such as those involved in the domestication of food crop species.

### 1.2. Seed Dormancy and Germination in Crop Domestication: Some Highlights

Plant domestication is a co-evolutionary transformation process in which wild plants evolve into crop plants through natural or artificial selection, adapting to human agroecological niches and preferences [27]. While crop domestication has been essential for historical development, it has also led to a significant reduction in biodiversity. The process of domestication of plants and animals for food production began globally approximately 12,000–11,000 years ago. This gradual transformation of plants over extended periods of domestication, marked by gene flow with wild relatives, was a pivotal step in the emergence of agriculture [28,29]. During domestication, both yield and harvestability were enhanced concurrently. Since then, crop improvement efforts have predominantly concentrated on increasing yield. However, many crops experienced greater biomass loss from herbivory compared to their wild relatives, mainly due to reduced defense mechanisms that arose during domestication [30,31]. Genetic research is increasingly identifying domestication genes, especially in plants [32]. Several researchers have proposed that key crop species emerged through multiple independent domestication events from their wild relatives. However, the multiple-origins hypothesis for maize and rice has been challenged by several authors [33,34,35]. Rice is one of the oldest domesticated crops, having been cultivated for approximately 11,000 years [36]. The domestication of rice involved numerous changes, including modifications in awn development, seed shattering, grain quality, plant architecture, grain size, heading date, panicle size, and seed dormancy. Interestingly, Choi and Purugganan (2018) confirmed that de novo domestication likely occurred only once, with domestication alleles subsequently transferred between rice sub-populations through introgression [37]. Recently, the QTL locus *KRN2* in maize and its ortholog in rice, *OSKRN2*, have been identified. Both loci underwent convergent selection, where unrelated species develop similar traits due to adaptation to similar environments, during their domestication processes. Evolutionary and functional evidence of convergent selection on *KRN2/OsKRN2* for grain number in both maize and rice has been demonstrated by Chen et al. (2022) [38]. Furthermore, a complete loss-of-function allele of *KRN2/OsKRN2* has been shown to increase grain yield without apparent negative impacts on other agronomic traits. *KRN2/OsKRN2* encodes a WD-repeat (WD40) protein, a family known for its role in plant abiotic stress response [38]. It interacts with a gene of unknown function, *DUFF1644*, to negatively regulate grain number in both crops [39,40,41].

Through artificial selection and domestication, humans have aimed to develop cereal crop species with lower seed dormancy compared to their wild ancestors, reduced seed shattering, larger seed size, and more synchronous germination, all to enhance resource availability and agricultural efficiency [42,43]. This applies to maize, wheat (the first domesticated staple food crop), rice, barley, beans, sorghum, and rye as well [44,45,46]. While seed dormancy offers advantages in wild ecosystems, it is generally deemed undesirable in crops due to its potential to reduce yield and/or quality. In seed crops, the loss of seed dormancy is often one of the first traits selected during the initial stages of domestication [47]. Physical seed dormancy is a significant trait in the domestication of many legume species. The development of water impermeability in the seed coat serves as a mechanism for physical dormancy [48]. Interestingly, recent findings suggested that the domestication of the common bean (*Phaseolus vulgaris*) exerted considerable selection pressure favoring rapid seed water uptake. This process likely induced significant changes in allele frequencies, particularly affecting the non-functional variant of an ortholog of pectin acetylesterase-8 (*PAE8*) [44]. *PAE8* is the primary pectin acetylesterase gene expressed in the developing seed coat of common bean. The presence of a non-functional *pae8* allele has been linked to an enhanced rate of water absorption and an increased percentage of germination in aged seeds [49]. Indeed, a 5-bp insertion in an ortholog of *PAE8* is likely a significant causative mutation responsible for the loss of seed dormancy during domestication. Recent studies have confirmed that *PAE8* plays a crucial role in seed imbibition, although other genes, such as those involved in seed coat coloration, may also contribute to this trait [49]. This latter process will be discussed further later on (see Section 3).

On the other hand, genetic data suggest that the process of domestication may have occurred in multiple stages throughout history [47,50]. That is, during domestication, depending on the species, humans selected for traits such as reduced seed dispersal ability without human intervention, fewer unproductive side-shoots, decreased seed dormancy, larger seeds, more predictable and synchronous germination, and in some seed-propagated species, larger and more prominent inflorescences. However, humans did not address all these traits simultaneously; instead, they distinguished between traits that underwent early selection (domestication genes) from those that were selected later to develop diversified and improved crops (improvement genes) [32].

**Figure 1:**
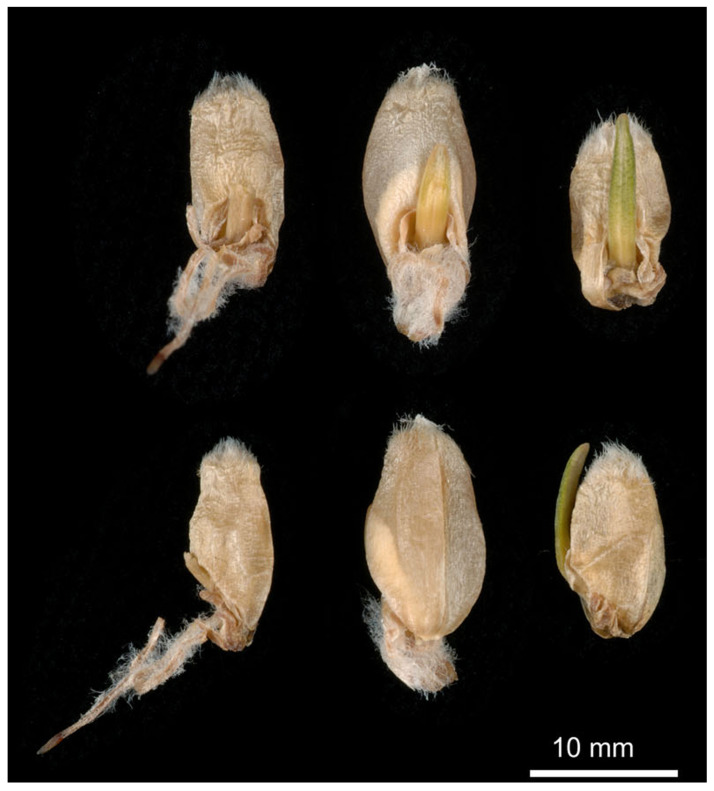
Wheat seeds exhibiting pre-harvest sprouting (courtesy of J. Barrero-Sánchez; https://people.csiro.au/B/J/Jose-Barrero (accessed on 25 May 2024 )).

**Figure 2:**
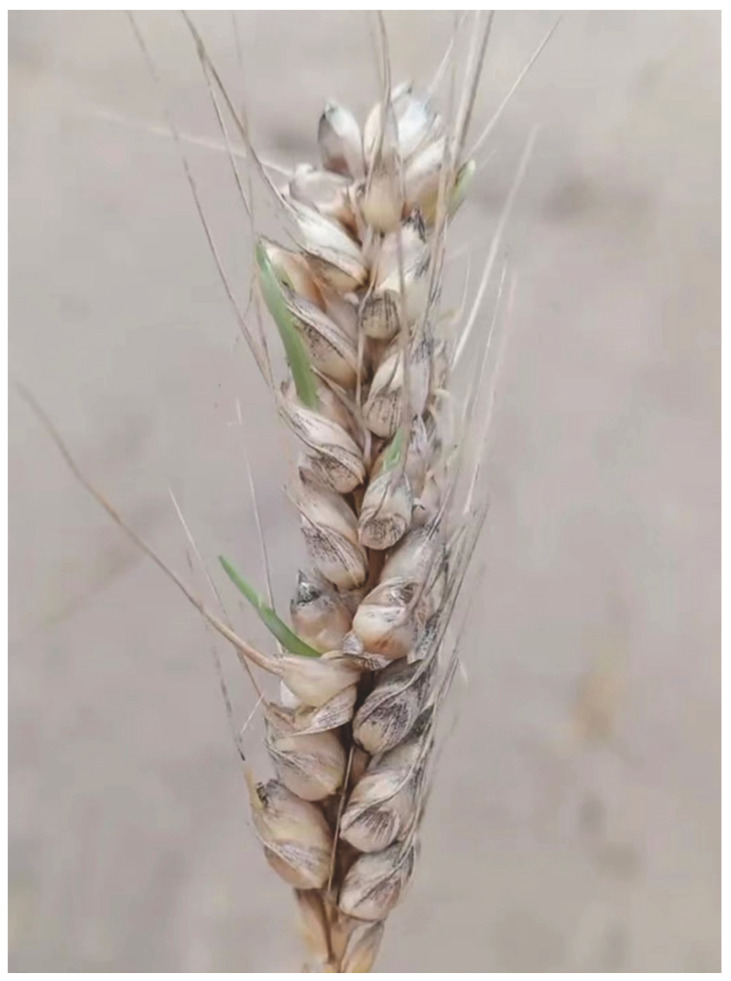
Wheat spike with pre-harvest sprouting (courtesy of Z. Pang and Y. Liang; ycliang@zju.edu.cn).

In summary, the domestication process aimed to produce crop species with weak seed dormancy intensity to provide goods and services useful to humans [51,52,53,54,55,56]. While reducing seed dormancy intensity initially seems highly beneficial for rapid and synchronized germination across various environmental conditions, it also presents certain drawbacks. Throughout domestication and breeding, the selection for rapid and uniform germination has decreased seed dormancy, thereby increasing the susceptibility of cereal plants to pre-harvest sprouting (Figure 1 and Figure 2), a central issue addressed in this update. Recently, Maeda and Yamaguchi (2017) conducted an interesting study on seed dormancy during crop domestication, focusing on the evolutionary changes in seed dormancy traits [57]. Their study offers valuable insights into how human intervention through domestication has shaped the dormancy traits of modern crops, carrying significant implications for agriculture and the sustainability of food production systems. 

### 1.3. Agricultural and Economic Losses Caused by Pre-Harvest Sprouting 

In regions where rainfall during the harvest season is common, pre-harvest sprouting damage is frequently reported. This premature germination can compromise the quality of the grain, making it unsuitable for many end uses. In other words, pre-harvest sprouting has emerged as a significant challenge for global agricultural production, leading to diminished grain quality, reduced yields, and lower commercial value. This results in substantial annual economic losses amounting to billions of dollars worldwide. Starch degradation (see further details below regarding α-amylase) due to pre-harvest sprouting can reduce grain quality and crop value by up to 30%. In severe instances, grains may become unsuitable for human consumption and are consequently relegated to animal feed. The economic impact, coupled with the need for stable food production, underscores the importance of identifying mechanisms to control pre-harvest sprouting and developing resistant cultivars. Ongoing efforts to mitigate pre-harvest sprouting are crucial for enhancing food security and ensuring the sustainability of cereal production systems worldwide. Despite its significant impact, the molecular mechanism responsible for pre-harvest sprouting remains largely unknown. Therefore, it is essential to elucidate the physiological and molecular mechanisms of pre-harvest sprouting to prevent this event. Developing and utilizing pre-harvest sprouting-resistant varieties is currently the most effective solution to address this problem. Although advancements in breeding programs have improved tolerance to pre-harvest sprouting in two notable cereal crops, barley, and wheat, newer varieties remain susceptible.

Cereals are the most important crops worldwide, with an annual production exceeding 2788 million tons. Rice (*Oryza sativa*) is a primary and stable food source for more than half of the world’s population. Pre-harvest sprouting is a detrimental phenomenon that frequently occurs in rice-growing regions with high temperatures and precipitation during rice grain maturation (rainfall exceeding 100 mm per hour). Specifically, pre-harvest sprouting affects more than 6% of conventional rice planting areas in the Yangtze River Basin and South China each year. The incidence of pre-harvest sprouting is known to increase primarily after the yellow-ripe stage of rice grain filling. This increase is thought to be influenced by the steady decrease in ABA content, which drops from its peak during grain development until maturation. Pre-harvest sprouting varies according to the number of days after heading, the position of seeds on the panicle, and the dormancy characteristics of different rice varieties. Rice develops the potential to sprout during the late grain-filling stage, particularly after a certain period has elapsed since grain-filling was completed. At this stage, more than 50% of rice grains can be susceptible to sprouting.

Common wheat (*Triticum aestivum*), which is a staple food around the world and the second most cultivated crop worldwide, feeds around 40% of the world’s population, providing more than 20% of the calories and proteins consumed globally by humans. Pre-harvest sprouting is one of the climatic disasters affecting the safety of wheat production worldwide and is responsible for up to USD 1 billion in annual losses. The annual financial loss of Canadian wheat alone from pre-harvest sprouting is estimated to exceed USD 100 million. These losses directly impact global food security. In China, about 25 million hectares of wheat are affected by pre-harvest sprouting each year, accounting for 83% of the whole wheat-growing area. However, farmers and the flour processing industry in China prefer white-grained varieties because of their high flour yield, more efficient flour extraction, high ash content, better appearance, and less bitter taste in the final product. Compared to chemical control, using varieties with high resistance to pre-harvest sprouting is more economical, safer, and effective in reducing damage. Pre-harvest sprouting is notably more severe in white-grained wheat than in red-grained wheat. In regions like China, where farmers prefer white grains for their higher flour yield, it is essential to develop and cultivate white-grained varieties that are resistant to pre-harvest sprouting. This requires an in-depth understanding of the genetic basis of both grain color and sprouting resistance, as well as the relationship between these two traits.

In the Cucurbitaceae family, cucumber (*Cucumis sativus*), a major vegetable crop with significant economic and biological value, faces a critical problem with pre-harvest sprouting. China is the leading producer of cucumbers, with its cultivation area accounting for 54% of the world’s total (1,985,000 hectares globally in 2018. Pre-harvest sprouting can reduce cucumber seed yield by up to 10–30%, depending on the severity of the sprouting and environmental conditions. It can also lower seed germination rates by 20–50%, significantly impacting seed viability and quality. In severe cases, economic losses due to pre-harvest sprouting can amount to thousands of dollars per hectare.

Barley (*Hordeum vulgare*) is the fourth most produced cereal worldwide and is highly susceptible to pre-harvest sprouting damage. This susceptibility leads to significant losses, especially in regions with substantial rainfall near harvest time. Pre-harvest sprouting can reduce barley yields by up to 10–20%. Sprouted barley grains typically have diminished quality, impacting their market value. This is particularly problematic in the brewing industry, where high-quality malt is essential. Sprouting causes the starch in the grain to convert into sugars, negatively affecting the malting process. Consequently, the suitability of barley for brewing and other industrial uses is reduced. Additionally, barley affected by pre-harvest sprouting often requires extra processing to remove or sort out lower-quality grains, leading to increased operational costs and decreased profitability. In severe cases, the economic losses can reach thousands of dollars per hectare.

Together, by integrating advancements in breeding techniques, effective management practices, and timely harvesting strategies, it is possible to mitigate the effects of pre-harvest sprouting. This approach can enhance the economic viability and overall productivity of crop production. However, the complete or partial eradication of pre-harvest sprouting in crop species remains elusive. Despite ongoing efforts, achieving full control over this issue continues to be a significant challenge. Finally, it is important not to overlook that poor-quality foods derived from pre-harvest sprouted grains can likely have significant implications for human health. 

## 2. Interplay between ABA, GAs, and α-Amylase in Regulating Pre-Harvest Sprouting

Although domesticated reproductive units, such as seeds from crop species, can germinate very quickly and uniformly, they are also highly susceptible to pre-harvest sprouting [26,58]. The major factors influencing the pre-harvest sprouting include environmental conditions, seed dormancy, seed coat color and permeability, α-amylase activity, endogenous hormones levels, multigenic control, germination inhibitors in glumes and testa, QTL, and other factors [59]. The problem of pre-harvest sprouting, particularly in food crops, can be confounded with others known as late maturity alpha-amylase (LMA) or vivipary (see below). Precocious germination, termed vivipary, takes place during the maturation phase of grain development. Vivipary occurs on the mother plant under high humidity; so viviparism can also be induced by fungal pathogens such as Fusarium [60,61]. That is, high humidity creates a favorable environment for saprophytic fungi infection. Pre-harvest sprouting is partly genetically controlled with many QTLs related to pre-harvest sprouting or seed dormancy identified in rice [56,62]. However, only a few genes associated with pre-harvest sprouting have been isolated thus far. Interestingly, vivipary disrupts the replenishment of seed banks (natural repositories of seeds often in a dormant state) thereby affecting the preservation of genetic diversity in soils. Pre-harvest sprouting arises from insufficient seed dormancy (weak dormancy) and occurs when rain and/or prolonged wet conditions prevail in the environment surrounding a domesticated plant during grain maturation and ripening (Figure 1 and Figure 2). Alternatively, this sprouting can occur in mutant seeds with low levels of ABA [4,63,64]. By contrast, seeds with strong dormancy are less likely to germinate before harvest and therefore are more resistant to pre-harvest sprouting [63,65]. As previously noted, domestication makes seeds of the mother plant more susceptible to pre-harvest sprouting.

Regarding starch synthesis, strong correlations have been observed between ABA concentrations, grain filling, and the capacity for starch synthesis in developing grains of three notable cereals: wheat, barley, and rice [66,67,68]. Adverse weather conditions may also directly reduce the rate of grain filling, potentially leading to the loss of embryo dormancy and pre-harvest sprouting. To explore whether grain filling influences embryo dormancy and susceptibility to pre-harvest sprouting, Howard’s group studied a collection of barley mutants with impaired starch synthesis [55]. The mutant lines showed reduced dormancy and increased pre-harvest sprouting in the field compared to the parental lines. This study established a connection between grain filling, the development of grain dormancy, and the incidence of sprouting. The findings clearly indicate that impaired starch synthesis in developing barley grains heightens susceptibility to pre-harvest sprouting. The observed decrease in dormancy in low-starch lines does not seem to be related to changes in ABA content but may instead be due to reduced sensitivity to ABA. Overall, these results suggest that the development of dormancy in barley grains is significantly influenced by the rate of starch accumulation in the endosperm [55].

Hormonally speaking, ABA is a well-known hormone that induces seed dormancy and helps maintain mature seeds in a dormant state [12,69]. ABA is sensed by the pyrabactin resistance 1 (PYR1)/PYR1-like (PYL)/regulatory components of the ABA receptor family of proteins [70,71]. Resistance to pre-harvest sprouting is typically linked to increased seed dormancy. In mature seeds, higher levels of ABA or enhanced ABA signaling promote dormancy, reducing the likelihood of vivipary. Thus, managing ABA concentrations and signaling during seed maturation is a key strategy in developing crop varieties with enhanced resistance to pre-harvest sprouting [4,72,73,74,75]. Rice PYLs have similar functions to Arabidopsis PYLs in promoting seed dormancy [58]. Interestingly, the highest frequency of pre-harvest sprouting was observed in *pyl1-6* and *pyl12* rice mutants. Specifically, these mutants exhibited significantly higher frequencies of pre-harvest sprouting compared to the WT, suggesting that PYL1 and PYL12 play crucial roles in establishing seed dormancy [58]. Surprisingly, PYL12 cannot bind to ABA in in vitro assays. Although ABA/GAs is a key factor involved in the regulation of seed dormancy and resistance to pre-harvest sprouting, the specific role of GAs in pre-harvest sprouting remains elusive. Nevertheless, the grain yield modulator *miR156* regulates plant shoot architecture and grain size and controls seed dormancy through the GA pathway in rice [58]. Interestingly, enhancing seed dormancy through *miR156* mutations is not mediated by the ABA pathway, but rather by suppressing GAs biosynthesis and signaling, and promoting GAs deactivation. This group demonstrated an efficient and effective method to inhibit pre-harvest sprouting and increase seed longevity without compromising productivity through CRISPR/Cas9 mutagenesis of the *MIR156* gene [58].

Recent insights into the molecular mechanisms of vivipary in plants have primarily been derived from studies on cereals and mutants of Arabidopsis. Together, the reduction of ABA levels and the increase of GAs, brassinosteroids, auxins, and cytokinins in embryonic tissues are critical changes that determine viviparous germination. Recently, transcriptomic and metabolomic analyses of maize vivipary mutants suggest that ABA and GA biosynthesis and signaling are primarily responsible for vivipary [74,75].

The pre-harvest sprouting has become a significant problem for agricultural production worldwide, resulting in reduced grain quality, yield, and commercial value [76,77,78,79] (Figure 1 and Figure 2). Sprouting involves a sequential series of events starting with rain interception by the spike’s vegetative structures, followed by the transfer of water to the enclosed grain, germination, and the subsequent production of various hydrolytic enzymes. To date, the molecular mechanisms that enable domesticated seeds to detect specific hydration levels, which trigger germination and lead to sprouting, remain unknown. This results in substantial annual economic losses amounting to billions of dollars worldwide. Furthermore, poor-quality foods derived from pre-harvest sprouted grains can have significant implications for human health. 

Although a clear relationship exists between pre-harvest sprouting and ABA in seeds, the mechanism by which this hormone alters seed physiology in response to environmental factors that trigger vivipary remains unknown. In 2024, a notable study clarified certain aspects of this mechanism. Wang’s group identified a transcriptional regulator, *SLR1-like2* (*SLRL2*), and demonstrated that it is the key gene mediating the regulation of ABA on rice quality [80]. *SLRL2* overexpression has been shown to potentially improve rice quality. In rice grains, the *Waxy* (*Wx*) gene is responsible for amylose synthesis, a crucial determinant of eating and cooking quality. Additionally, NF-YB1, a seed-specific nuclear factor, regulates the expression of sucrose transporters in the aleurone layer, facilitating sugar loading into the rice endosperm. Collectively, these findings from Wang’s group highlight important insights into rice quality enhancement [80]: (i) the overexpression of *SLRL2* increased pre-harvest sprouting resistance, whereas *SLRL2* mutation only slightly decreased rice resistance to sprouting. *SLRL2* regulates rice pre-harvest sprouting, at least in part, by modulating the expression of *MFT2*, a key positive regulator of ABA signaling and with seed-specific expression [81,82]; (ii) the interaction between SLRL2 and NF-YB1 can probably inhibit the degradation of NF-YB1; (iii) SLRL2 may bind directly to the *Wx* promoter and regulate its expression; (iv) SLRL2 also directly binds to *bHLH144*, an important gene that responds to *NF-YB1* stability, to promote and suppress its expression; and (v) NF-YB1 binds directly to the promoter of *SLRL2* and represses its transcription. Taken together, all these outstanding data indicated that *SLRL2* is a canonical TF with direct DNA binding ability and can repress the expression of both *bHLH144* and *Wx* genes, thereby modulating rice grain quality. The data above show that the key members of the identified NF-YB1-SLRL2-bHLH144 module are also involved in determining other seed-related growth and development events, including pre-harvest sprouting. All this indicates that this module is central to mediating ABA regulation of rice quality. In other words, this outstanding study revealed an ABA-responsive regulatory cascade that functions in both rice quality and seed dormancy [80]. On the other hand, it has also recently been described that the Seed Dormancy-6 (*SD6*) gene is a quantitative genetic locus encoding a bHLH TF [11]. It underlies natural variation in seed dormancy. *SD6* and another bHLH factor called ICE2 (Inducer of C-repeat binding factor Expression-2), act antagonistically in controlling seed dormancy by directly regulating the ABA catabolism gene *ABA8OX3* and indirectly the ABA biosynthesis gene *NCED2* via OsbHLH048. The weak dormancy allele of *SD6* is common in cultivated rice but experiences negative selection in wild rice. *SD6* is a valuable target for breeding to mitigate pre-harvest sprouting in cereals under field conditions [11]. 

There are two major, genetically distinct, causes of elevated α-amylase in cereal grain, pre-harvest sprouting and late maturity α-amylase (LMA) [83,84,85,86]. LMA is a genetically determined trait that involves the synthesis of α-amylase during late grain development (ripening) in the absence of preharvest sprouting, which occurs in mature grains. The wheat grain can germinate precociously (viviparism) during its maturation phase, a phenomenon associated with elevated α-amylase expression [87]. The Hagberg–Perten falling number (FN) assay, developed in the early 1960s, provides a rapid and cost-effective means of determining α-amylase activity (i.e., a low FN indicates elevated α-amylase activity) [88,89,90]. However, cultivars with high FN are not necessarily tolerant to pre-harvest sprouting, as FN does not account for all components of pre-harvest sprouting damage [91]. Along with the sprouting index, weighted germination index, and germination rate, these are the four major traits that characterize pre-harvest sprouting [58]. While both LMA (induction of α-amylase by cool temperatures) and pre-harvest sprouting (induction of α-amylase by rainfall) result in the expression of α-amylase in the aleurone layer, α-amylase is expressed randomly throughout the aleurone during LMA, resulting in similar levels at both the embryo-proximal and distal end of the grain. In contrast, pre-harvest sprouting results in higher levels of α-amylase at the embryo-proximal end of the grain [92]. Interestingly, the location of α-amylase expression in the grain can be used to distinguish between LMA and pre-harvest sprouting. A comprehensive understanding of LMA from the underlying molecular aspects to the end-use quality effects will greatly benefit the global wheat industry and those whose livelihoods depend upon it [86]. On the other hand, functional *Vp-1* was required for the full repression of germination-related α-amylase expression in aleurone cells. Regulation during LMA and pre-harvest sprouting might share molecular and genetic mechanisms [92,93]. At present, it is not known whether LMA can serve any beneficial function in native environmental grasses [86]. There is currently no molecular marker known to be uniquely present during LMA but absent during pre-harvest sprouting. Given all the above, while the issue remains a significant concern, current scientific efforts are underway to address and mitigate it. 

## 3. How Seed Color Affects Pre-Harvest Sprouting Resistance

As stated above, pre-harvest sprouting occurs due to the presence of weak seed dormancy traits in certain domesticated crops. Recently, research on pre-harvest sprouting has mainly concentrated on identifying individual genes that affect seed dormancy. However, only a few genes have been consistently validated across multiple studies as being associated with this phenomenon. In wheat, the genetic architecture influencing pre-harvest sprouting is governed by numerous genes with additive effects [94]. Genetic research has identified genes associated with seed dormancy and pre-harvest sprouting resistance, some of which are linked with seed coat color [25]. Seed color in the external cover is determined by pigments such as anthocyanins, flavonoids, and carotenoids, which also play roles in various physiological characteristics of the seed [95]. In wheat, red grain color is traditionally used as a marker for resistance to vivipary [96]. Studies over the past decade have shown that the red seed coat is associated with higher levels of seed dormancy and tolerance to pre-harvest sprouting compared to white-grained wheat [97]. However, the relation between grain color and dormancy remains unknown. In white-grained wheat, dormancy is conditioned by the cumulative effects of several QTLs that delay the onset of the capacity to germinate during ripening and after ripening [98]. Other significant genes, such as *TaMFT* (3A, 3B1, 3B2, 3D), also referred to as *TaPHS1* and belonging to a family of proteins known as phosphatidyl-ethanolamine-binding proteins (PEAPs), *AtABI3*, *OsABI5*, *AtDOG1*, *AtLEC2*, *OsVP1*, *OsNCED3*, *OsPHS9*, *OsPHS8*, and *ZmVP1-10*, have also been identified. These genes have been linked to various aspects of pre-harvest sprouting, including their roles in the biosynthesis, catabolism, perception, and signaling pathways of ABA and GAs [99].

As indicated above, grain color is the seed phenotype most classically associated with pre-harvest sprouting resistance. The red seed color is one of the most important traits for pre-harvest sprouting resistance in wheat. The red color (R) is controlled by the genes *R-3A1*, *R-3B1*, and *R-3D1*, located on chromosomes 3A, 3B, and 3D, respectively, and can enhance seed dormancy by increasing the sensitivity of embryos to ABA [100]. These genes are additive and have pleiotropic effects on pre-harvest sprouting. The effects of grain color on pre-harvest sprouting are believed to result from the accumulation of the red pigment precursor, catechin (proanthocyanidins) [101], which inhibits germination [100,102]. In wheat, *TaMFT* was cloned from the red-grained cultivar Zen [19], and the white cultivar Rio Blanco [21]. The red seed color controlled by an *R*-gene can promote pre-harvest sprouting resistance with strong seed dormancy in durum wheat [103]. To highlight, *R-1* genes encode MYB10 transcription factors (TF), which are also involved in both flavonoid biosynthesis and the ABA signaling pathway [100,104]. Red-grained wheat varieties are usually more tolerant to pre-harvest sprouting than the white-grained ones [100]. Resistance in many durum wheat genotypes, including red-seeded varieties, has been studied, and it was concluded that red-seeded wheat carrying the *R*-gene exhibits the strongest resistance among them [105]. Together, the introduction of an *R*-gene for red seed color, like QPhs-5AL from bread wheat, into durum wheat by interbreeding was very effective in introducing pre-harvest sprouting resistance into durum wheat [103]. The *R-1* gene encoding TaMYB10 TF was the most effective in promoting pre-harvest sprouting resistance. *TaDFR-B* is another structural gene involved in the production of red pigments (flavonoids) that affects pre-harvest sprouting resistance and seed color by controlling anthocyanin synthesis in red-grained Chinese wheat germplasm [106]. However, transient expression of *TaDFR-B* in coleoptiles of *T. aestivum* cv. Chinese Spring revealed that increasing *TaDFR* gene expression did not induce the synthesis of anthocyanins. This may be because the red pigment in wheat grain is synthesized by four enzymes in the flavonoid synthesis pathway, with DFR located downstream of the other three enzymes [106].

Finally, in the non-crop species *Sisymbrium officinale* (Brassicaceae), which produces seeds of heterogeneous color (i.e., black, gray, and very light), seed color strongly affects the degree of germination, and ethylene is heavily involved in the process [107]. However, pre-harvest sprouting has not yet been studied in *S. officinale* to determine whether it is affected by seed coat color. Later, Yan et al. (2023) demonstrated a significant correlation between the germination index and grain color in a set of 168 wheat varieties (lines) [108]. On the other hand, the search for genes controlling pre-harvest sprouting in a color-independent manner is of special interest [21,109]. It was recently suggested that novel genetic mechanisms exist to control pre-harvest sprouting resistance independently of color. In a study of a collection of wheat varieties, it was found that variation in coloration does not correlate with resistance to pre-harvest sprouting [110].

The findings from two independent studies made in wheat suggest that variation in pre-harvest sprouting resistance is mainly governed by the genotype. However, environmental factors during grain development, especially temperature, significantly affect the expression of seed dormancy [111]. Consequently, it is essential to investigate candidate genes that govern pre-harvest sprouting resistance across various germplasms to gain a comprehensive understanding of the genetic mechanisms at play. By studying and manipulating these genes, it is possible to develop crop varieties with improved resistance to pre-harvest sprouting, thereby enhancing the stability and quality of grain yields. These challenges are particularly significant due to the substantial global economic importance of crops such as wheat and barley. In other words, the majority of recent research on pre-harvest sprouting focuses on genetic studies of grasses with high socioeconomic importance worldwide. At the genetic level, common wheat is an allopolyploid species originating from hybridization between tetraploid *Triticum turgidum* and *Aegilops tauschii* in the Fertile Crescent environment, where little rain falls during the harvest season, and pre-harvest sprouting is, therefore, rare. *Aegilops tauschii* (i.e., goatgrass) shows a significant level of resistance to pre-harvest sprouting, a characteristic particularly valuable in agriculture. So, *A. tauschii* is a wild relative of wheat and has been identified as a source of several beneficial traits, including pre-harvest sprouting resistance. This resistance is crucial for developing wheat varieties that are more resilient to adverse weather conditions before harvest. Hybrids have been recreated by crossing *T. turgidum* with *A. tauschii* to produce so-called synthetic hexaploid wheat to analyze pre-harvest sprouting resistance.

As stated above, only a few functional genes have been identified for pre-harvest sprouting *resistance.* Thus, six genes controlling pre-harvest sprouting in wheat have been isolated by map- or homology-based cloning, including *TaVp-1* encoding a B3-domain TF [22,112], *TaMFT/TaPHS1* encoding PEAPs [19,21], *TaMKK3* encoding a mitogen-activated protein kinase kinase (MKK)-3 [18], *TaQsd1* encoding an alanine aminotransferase [113], *TaDOG1L1* [114] and *TaSdr* with unknown functions [115]. Later on, an in-depth update will be provided on *PHS1* and its causal gene *TaMKK3*, and *Pv-1*, two of the most extensively studied genes associated with pre-harvest sprouting. Very recently, the major QTL *SDR3.1* associated with rice seed dormancy was cloned [116]. This discovery could potentially lead to a better understanding and manipulation of seed dormancy traits in rice, which could have implications for agriculture and food security. The gene *SDR3.1* encodes a mediator of OsbZIP46 deactivation and degradation (MODD), which plays a role in negatively regulating seed dormancy by inhibiting the transcriptional activity of ABIs. In simpler terms, *SDR3.1* controls pre-harvest sprouting and suppresses the transcriptional activity of OsABI3 (a B3 domain transcription factor) and ABI5 (a basic leucine zipper protein) [116]. On the other hand, SDR3.1 is a nuclear protein rather than a TF, and evolutionarily, it serves an essential function in monocotyledons. The regulation of seed dormancy by *SDR3.1* relies more on its protein activity than its expression levels. As a result, *SDR3.1* has undergone intensive selection during the domestication and breeding of rice cultivars.

Meanwhile, Dr. Giroux’s research on sprouting in cultivated cereals uncovered the following insights in bread wheat and barley: (i) The research provided unique insights into the genetic mechanisms governing pre-harvest sprouting in bread wheat identifying specific loci on wheat chromosomes that are strongly linked to pre-harvest sprouting resistance [72,117,118,119]; (ii) controlled sprouting in wheat enhances the quality and consumer acceptability of whole wheat bread [117]; (iii) *HvAlaAT1* is strongly linked with dormancy in after-ripened grain, whereas *HvMKK3* shows a strong association with dormancy at physiological maturity. This suggests that different genes may be involved in regulating dormancy at different stages of grain maturity [18,19]; (iv) the research highlighted the genetic linkage between seed color and PHS resistance, emphasizing the importance of considering these traits in breeding programs [72]; (v) the homeologs *TAMFT-3A* and *TAMFT-3B2* are associated with pre-harvest sprouting in wheat [119,120]; (vi) unassessed durum wheat varieties were evaluated for susceptibility to pre-harvest sprouting. This study can provide a valuable resource for spring and durum wheat breeders aiming to select alleles of MFT (Mother of FT and TFL1) that influence susceptibility to pre-harvest sprouting [120].

## 4. Several Genes Associated with Pre-Harvest Sprouting in Crops

### 4.1. Pre-Harvest Sprouting-1 Locus (PHS1) and Causal Gene TaMKK3

Pre-harvest sprouting is a quantitative trait controlled by multiple genes and QTLs that are important for breeding pre-harvest sprouting-resistant varieties [64,78,121]. In wheat, more than 250 QTLs for pre-harvest sprouting resistance have been identified [59,122]. Although pre-harvest sprouting is a complex multigenic trait, a significant proportion of the natural variation for sprouting is controlled by a few major QTLs. Thus, a major QTL for seed dormancy has been detected in bread wheat. This QTL was designated as a major gene called *TaPHS1* [18,123]. Comparative genome analysis indicated that the *PHS1* region in wheat might correspond to the *SD2* locus in barley [124]. Likewise, an *MKK3* named *TaMKK3A,* located on chromosome 4A was identified as the causal gene for *TaPHS1*, providing direct evidence that *PHS1* in wheat is an ortholog of *SD2* in barley [18]. Concluding, *TaMKK3* was observed to be a major regulator of mature seed dormancy seeds [18]. Protein kinases are critical components in the phosphorylation of proteins and signal transduction pathways and were reported to be associated with ABA signaling [125]. The homology of *TaMKK3* to *MKK* genes in Arabidopsis suggests *TaMKK3* affects dormancy by positively modifying ABA responsiveness [126]. The identification of *MKK3* as the causal gene for *PHS1* means that variations in the *MKK3* gene sequence are responsible for differences in pre-harvest sprouting resistance. With the identification of *MKK3* as a causal gene, it becomes a target for genetic engineering approaches such as CRISPR/Cas9 to enhance pre-harvest sprouting resistance in wheat. The discovery by Torada et al. (2016) is especially significant given that wheat and barley are the most studied species concerning pre-harvest sprouting resistance [18]. The finding that *TaPHS1* is an ortholog of *HvSD2* in barley (*Hordeum vulgare*) implies that these genes share a common ancestor and perform similar functions in different species. Orthologous genes can be exploited to transfer knowledge from model organisms to crops. Interestingly, recognizing *PHS1* as an ortholog of *HvSD2* in barley means that barley research can directly inform wheat breeding strategies, accelerating the development of resistant varieties. *TaMKK3A* is a candidate for the seed dormancy locus *PHS1-4AL* on chromosome 4 of wheat [18,123]. Similarly, Nakamura et al. (2018) conducted significant work on pre-harvest sprouting resistance in barley, further supporting the cross-species insights between wheat and barley [17]. Strikingly, complementation analysis showed that the transformation of a dormant bread wheat cultivar with the *MKK3* gene clearly reduced seed dormancy, providing functional evidence of *MKK3’s* role in controlling pre-harvest sprouting [18]. On the other hand, *TaMKK3A* was independent of seed coat color and is also orthologous to a rice gene on chromosome 6. Identification of *MKK3* as the causal gene for *PHS1* by Torada et al. (2016) represents a significant advance in understanding the genetic control of pre-harvest sprouting resistance in wheat. Finally, Torada et al. (2016) discovery, along with the earlier work by Liu et al. (2013) on the cloning of *TaPHS1* from the 3AS QTL Qphs.pseru-3AS, not only provides clear targets for breeding programs but also enhances understanding of the genetic relationships between wheat and barley [21]. *TaMFT-3A*, a positive regulator of ABA sensitivity identified in 2011, is a highly valuable pre-harvest sprouting resistance gene for breeding white wheat cultivars [21,127,128,129]. Multiple *TaMFT-3A* mutations have been associated with either pre-harvest resistance or susceptibility [21,129]. As indicated in this update, resistance to pre-harvest sprouting is multi-genic, although a significant proportion of the variation in sprouting resistance among modern wheat cultivars is controlled by a few major QTL, including Phs-A1 on chromosome arm 4AL. Despite its importance, little is known about the physiological basis and the gene(s) underlying this crucial locus. Shorinola et al. (2016) characterized *Phs-A1* and demonstrated that it confers resistance to sprouting damage by affecting the rate of dormancy loss during dry seed after ripening. This study suggests the possibility that more than one causal gene underlies this major pre-harvest sprouting locus *Phs-A1* and provides a clear target for breeding programs [130]. Furthermore, wheat genes *Plasma Membrane 19* (*TaPM19-A1* and *TaPM19-A2*) [131] and *Seed Dormancy* (*TaSdr*) [23] were identified to play critical roles in the regulation of seed germination.

### 4.2. Viviparous-1 (Vp-1) Gene

Cereal VP1 proteins belong to a subfamily of B3 TFs controlling seed gene expression [132]. HvGAMYB (a transcriptional activator of seed storage protein in barley) and HvVP-1 proteins interact in vivo [133]. The B3 genes belong to a superfamily that encodes plant-specific TFs found from unicellular green algae to eudicots [134,135,136]. They all have at least one region known as the B3-binding structural domain, approximately 110 amino acids in length. The *B3* superfamily is composed of four subfamilies, including RELATED TO ABI3/Vp-1 (RAV) [132]. The third basic region of Viviparous-1 (Vp-1) in maize (*Zea mays*) is where the structural domain is first identified [137]. Two main functions for Vp-1 protein in seed development have been well documented: (i) activator of various seed-specific ABA-regulated genes such as late embryogenesis abundant (LEA) [138,139]; and (ii) transcriptional repressor of germination-specific genes such as α-amylase in the aleurone of maize endosperm [140,141]. Likewise, it was also demonstrated that α-amylase expression in aleurones is enhanced in developing embryos homozygous for *vp1*, which blocks ABA signal transduction, or for *vp5*, which blocks ABA synthesis [141].

The *Vp-1* gene encodes a dormancy-related TF in maize and is a major regulator of late seed development in both maize and wheat [138] and has been cloned and sequenced from several plant species. Molecular genetic studies revealed that *Vp-1* is important for the completion of seed maturation [142]. McKibbin et al. (2002) reported that mis-splicing of the pre-mRNA of *TaVp-1* occurred around the B3 domain, resulting in the production of truncated mRNAs and the generation of stop codons in the ORF [143]. That is, mis-splicing of *TaVp-1* transcripts led to reduced Vp1 protein production and malfunction of the gene, and diminished pre-harvest sprouting tolerance. Many major and minor genes, therefore, can impact tolerance to pre-harvest sprouting in diverse genetic backgrounds. Such mis-splicing was also detected in the majority of *TaVp-1* transcripts from diploid and tetraploid ancestral wheat, suggesting that modern wheat species have inherited the structure of *TaVp-1* from their ancestral species [143]. On the other hand, Fakthongphan (2016) used combining ability analysis for both pre-harvest sprouting tolerance and falling number from field-grown samples, to identify red wheat genotypes capable of donating genes, independent of seed coat color, which could improve pre-harvest sprouting tolerance of hard white wheat [109]. During the late stages of seed development, this organ undergoes maturation, acquires desiccation tolerance, and achieves primary dormancy [4]. Along with ABA and other factors, *Vp-1* is required for the regulation of these processes. In other words, *Vp-1* is a key component of ABA signaling and is required for the expression of the maturation program [132,144,145]. That is, *TaVP1 Vp-1* is a key component of ABA signaling and is required for the expression of the maturation program [144]. *The vp-1* gene from maize has a central role in pre-harvest sprouting, regulation of seed maturation, and repressing germination. As a regulator of the *C1* gene, which encodes a TF of the MYB family involved in the anthocyanin biosynthesis pathway, *vp-1* mutants result in an unpigmented aleurone cell layer and the seeds precociously germinate due to the reduced ABA sensibility [133,146,147].

Specific genes and enzyme activities have been identified that are activated or repressed by Vp-1 in vivo. Orthologues of *Vp-1* in Arabidopsis (*ABI3*) and other cereals (*HvVP1* in barley; *TaVP1* in wheat, etc.) have been characterized as essential regulators of the genes that encode storage proteins and LEAs during maturation [133,134]. However, the function of expressed Vp-1 mRNA at the early stage of zygotic embryogenesis [148], remains unknown. Vp-1B, a regulatory gene located on chromosome 3B, has previously been proven to be involved in inducing grain dormancy of wheat [149]. Vp-1 regulates transcription by activating the expression of ABA-inducible genes, mediated by indirect interaction with ABA response elements (ABREs) via bZIP TFs [150]. The maize Vp-1 transcript is induced by ABA [151]. Inactivation of the Vp-1 locus leads to disruption of seed maturation, resulting in vivipary. That is, the vp-1/abi-3 mutants produce seeds with reduced sensitivity to ABA, which fail to undergo normal maturation and germinate viviparously [152,153]. Thus, maize vp-1 mutant seeds are characterized by their incapacity to enter dormancy [134]. Analysis of *vp-1* mutants has shown that this locus performs two important functions: promoting seed maturation and repressing seed germination [154]. So, functional *VP-1* was required for the full repression of germination-related α-amylase expression in aleurone cells [141,153]. Analysis of an ABA deficient *vp-1 vp-5* double mutant indicates that ABA synthesized in the embryo interacts additively with Vp-1 to prevent precocious induction of α-amylase genes in the aleurone of the developing seed. These authors hypothesize that among the set of factors regulating α-amylase genes in developing aleurone cells, ABA and *Vp-1* appear to be relatively more important than GA synthesis or sensitivity. *Vp-1* action in this context is largely independent of the two hormones [141]. However, this aspect should be predominant to study in the future.

In barley, *HvVP1* and *HvFUS3* transcripts are abundant in both endosperm and embryo during seed development. In *Zea mays*, ZmVP-1 controls the *C1* gene that encodes a MYB-like TF and is a key regulator of the anthocyanin biosynthesis pathway in corn [155]. *VP-1* is the major AFL TF associated with the repression of genes encoding hydrolytic enzymes (i.e., α-amylase and proteases), which are involved in the storage mobilization of cereal seeds during post-germination. This repression was first described in *Z. mays* and later in *T. aestivum*, *A. fatua,* and *O. sativa*, and has been associated with the avoidance of pre-harvest sprouting.

The experiment in which transgenic wheat plants containing a functional *Vp-1* TF gene from *Avena fatua* were shown to exhibit reduced susceptibility to sprouting is striking. This assay suggests that *Vp-1*, a TF previously shown to repress germination in maize [138], may provide a transgenic route for reducing pre-harvest sprouting susceptibility [143]. This transgenic plant showed increased dormancy and tolerance to pre-harvest sprouting. Recently, *Vp-1*, orthologous of *ABI3* from *A. thaliana*, was characterized in *Hordeum vulgare*. *HvVP-1* is a transcriptional repressor of maturation and germination seed genes in barley [133]. Given that *HvVp-1* is expressed in the starchy endosperm and aleurone layer would favor the repression of the α-amylase and thus avoid the preharvest sprouting [133]. *HvVP1* not only prevents the premature expression of post-germination genes (encoding α-amylases, proteases, etc.) but also ensures that its disappearance during post-germination is required for the mobilization of reserves to take place. The transcriptional repression of hydrolase genes mediated by *VP1/ABI3* during germination has been previously reported in both monocot and dicot seeds, including species such as maize, tomato (*Solanum lycopersicum*), and *Arabidopsis thaliana* [156]. All data of this work demonstrate the participation of *HvVP1* in antagonistic gene expression programs and support its central role as a gene expression switch during barley seed maturation and germination.

The majority of pre-mRNAs transcribed from the *TaVp-1* homoeologues were spliced incorrectly, generating a large proportion of non-functional proteins that might have contributed to the increased pre-harvest sprouting [143]. The ability of Minamino, a highly dormant cultivar of hexaploid wheat, to accurately splice the majority of TaVp1 pre-mRNAs appears to be a specific trait that has been developed through breeding processes. The majority of TaVp-1, particularly TaVp-B1, are properly spliced and may function as TFs that play an important role in seed dormancy [157]. TaVP-B1 suppresses α-amylase expression and potentially regulates this suppression through interactions with GA-induced TFs or by directly influencing their expression [157].

In the past decade, Huang et al. (2011) demonstrated that transgenic wheat expressing an *AsVp-1* gene from wild oats displayed reduced germination, increased responsiveness to ABA, and enhanced tolerance to pre-harvest sprouting [158]. Molecular characterization and comparative analysis have shown that ZmVp-1, encoding a TF primarily involved in ABA signal transduction pathways, regulates vivipary and is associated with pre-harvest sprouting in wheat and other cereals. Moreover, expressing a *ZmVp-1* coding sequence with its native promoter in a specific wheat variety through *A. tumefaciens* transformation led to stable enhancement of seed dormancy and increased tolerance to pre-harvest sprouting. This transformation also resulted in reduced α-amylase activity, illustrating effective functional compensation by the foreign *Vp-1* gene [158]. It is noteworthy to highlight this year’s research on *TaVP-1*, particularly regarding the *TaSRO1* gene, which exhibits high expression in wheat seeds [159]. TaSRO1 interacts with TaVP-1 to modulate seed dormancy and pre-harvest sprouting resistance in wheat [160]. This recent 2024 study have several noteworthy implications: (i) despite the occurrence of mis-splicing in the majority of TaVP-1 transcripts, functionally intact TaVP-1 transcripts still play a crucial role in maintaining seed dormancy; (ii) TaVP-1 regulates wheat seed dormancy either through the embryo or the scutellum; (iii) TaVP-1 functions as a positive regulator, while TaSRO-1 acts as a negative regulator of ABA signaling; (iv) *TaVP-1* is epistatic to *TaSRO-1* in regulating pre-harvest sprouting resistance and ABA signaling; (v) TaSRO-1 physically interacts with TaVP-1 and suppresses the transcriptional activation of genes related to pre-harvest sprouting resistance; (vi) TaSRO-1 suppresses the transcriptional activity of the TaVP-1-TaABI5 module on genes related to pre-harvest sprouting resistance; (vii) mutants of *tasro-1* showed strong seed dormancy and increased resistance to pre-harvest sprouting, whereas mutants of *tavp-1* exhibited weak dormancy; (viii) biochemical evidence indicates that TaSRO-1 inhibits the transcriptional activation of TaVP-1 on genes associated with pre-harvest sprouting resistance, thereby promoting seed dormancy [160]. In summary, the introduction of *Vp-1* genes from one species into another of agronomic importance is increasingly significant. The study of transformant plants at genetic and physiological levels is crucial for understanding the mechanisms behind pre-harvest sprouting. This research underscores the importance of genetic manipulation in enhancing resistance to pre-harvest sprouting, thereby impacting agricultural practices (see [18,103,158]). Therefore, this research protocol should be continued throughout this decade, which is crucial for improving crop yield and quality in agriculture. 

### 4.3. Myb10/Tamyb10, R-1, and TaLTP2.128 Genes

Although a number of QTLs and genes related to pre-harvest sprouting in cereals have been reported, the molecular mechanisms underlying pre-harvest sprouting remain largely elusive. To contribute to this advancement, T*aMyb10-D* was identified as *PHS-3D*, playing a crucial role in pre-harvest sprouting resistance in wheat [104]. This seed-specific gene, previously known as *Myb10* or *R-1*, has pleiotropic effects and regulates flavonoid biosynthesis by activating the expression of various biosynthetic pathway genes such as PAL, FLS, CHS, CHI, F3H, GT, DFR, ANS, F3’H, and UFGT [104,161]. Specifically, the production of red pigments is regulated by *R-1*. The *TaMyb10* gene, *Myb10-D*, is highly expressed in wheat seeds (at 2 days post-anthesis, DPA) and in the aleurone layer (at 12 DPA). *Myb10-D* and its nuclear TF, Myb10-D, specifically contribute to pre-harvest sprouting resistance by activating the promoter of 9-cis-epoxycarotenoid dioxygenase (NCED) and functionally activating *NCED* expression. This promotes ABA biosynthesis during early seed development stages, thereby repressing germination [104]. Additionally, Myb10-D enhances flavonoid synthesis, leading to the accumulation of anthocyanins, which can hinder moisture and O2 exchange between the grain and the external environment, thereby impairing seed germination [162]. The findings of Lang et al. (2021) support that Myb10-D plays a critical role in promoting ABA synthesis, which suppresses germination by positively regulating NCED transcription [104]. Thus, Myb10-D can regulate both grain color and seed germination in wheat. Understanding and manipulating these genes through advanced breeding techniques can lead to the development of wheat varieties with improved resilience and quality, contributing to more stable and productive agricultural systems. In 2024, interesting work was published demonstrating that the combination of *TaMyb10* homologs is significantly associated with grain color and germination percentages. Accessions exclusively harboring *TaMyb10-D* displayed red seed color and reduced germination percentages, indicating the predominant role of *TaMyb10-D* compared to *TaMyb10-A* and *TaMyb10-B* [163]. Recently, Zhu et al. (2023) conducted a study using CRISPR/Cas9 to restore the Tamyb10 gene in wheat to create pre-harvest sprouting-resistant red wheat [164]. This group successfully converted a white wheat variety into a red one and improved its pre-harvest sprouting tolerance. In parallel, Zhu’s lab also presents an alternative strategy for crop improvement by editing genes that were lost through domestication via the restoration of reading frames. Additionally, it was recently demonstrated that Tamyb10 regulates dormancy release by modifying TaLTP2.128, a member of non-specific lipid transfer proteins, which acts as a trigger for germination. R-1 not only regulated grain color but also modified the expression of *TaLTP2.128* in imbibed grains. *TaLTP2.128* was expressed at higher levels in white grains (weak dormancy) than in red grains (strong dormancy), and its transcription increased with grain imbibition. Interestingly, the expression of *TaLTP2.128* was detected before germination “*sensu stricto*”, suggesting that this gene might act as a trigger of a transition from the dormancy stage to the germination stage [165]. In both these studies, grain color and dormancy were modified in transgenic lines into which *Tamyb10-D1* had been introduced [102,106]. On the other hand, the tissue sensitivity to ABA might be more important than its endogenous content in the R-1 regulation pathway, as the R-1 regulation of grain dormancy operates in an ABA-dependent manner. In summary, the results presented in this section contribute to advancing our understanding of resistance to pre-harvest sprouting in cereals and open new avenues for mitigating this detrimental issue for agricultural production. 

## 5. What Does the Future Hold for Managing and Warning about Pre-Harvest Sprouting?

The future will aim to harness advanced technologies and scientific progress to enhance the accuracy and timeliness of predictions. Advances in genetic and biotechnological research may lead to the development of crop varieties that are more resistant to pre-harvest sprouting. Understanding the genetic factors that contribute to sprouting can aid in breeding more customizable crops. Future efforts will focus on comprehensive gene function studies, practical field validations, and collaborative initiatives to enhance the ability to adapt to adverse situations with positive results. and productivity of crops.

Throughout this and other updates, it has been well established that pre-harvest sprouting is closely related to the seed dormancy process. This event is triggered at the end of zygotic embryogenesis and is regulated by signaling pathways in which ABA and GAs play a very active role. However, the precise mechanism driving this signaling puzzle remains far from understood. Nevertheless, the pre-harvest sprouting occurs when the seeds of the mother plant, with low dormancy intensity at physiological maturity, are exposed to an environment with high humidity and heavy rainfall. In studied crop species, sprouting involves a sequential series of events starting with rain interception by the spike’s vegetative structures, followed by the transfer of water to the enclosed grain, germination, and the subsequent production of various hydrolytic enzymes. To date, the molecular mechanisms enabling domesticated seeds to detect specific hydration levels that trigger germination and lead to sprouting remain unknown. In other words, water appears to be the trigger or facilitator of this detrimental event for human-utilized crops. There is no well-founded research demonstrating how endogenous water causes alterations in the pre-harvest sprouting mechanism in seeds with low ABA levels or non-deep dormancy. It seems clear that seeds with deep dormancy do not tend to exhibit pre-harvest sprouting. Unfortunately, during domestication, rice seed dormancy has largely been lost. Therefore, it is necessary to identify rice cultivars that have longer seed dormancy and clone seed dormancy genes to improve rice yield.

In summary, future research should focus on uncovering the specific points where water exerts an action that alters the complex puzzle of the ABA mechanism and its mode of action in mature seeds. An interesting protocol might be the detailed study of hydration relative to the hormonal sensitivity threshold and why the pre-harvest sprouting process, once triggered, does not reverse. Another practical problem concerns the identification of suitable genes for sprouting resistance and their introduction into current breeding material. Finally, it is also intriguing to study pre-harvest sprouting in species not integrated into crops, as well as the progression of this negative event throughout the evolution of plants that have not undergone domestication. Consequently, the future of pre-harvest sprouting research appears to be long and complex.
